# Morphologic Features of the Distal Femur and Proximal Tibia: A Cross-Sectional Study

**DOI:** 10.7759/cureus.12907

**Published:** 2021-01-25

**Authors:** Aditi Chaurasia, Ankita Tyagi, John A Santoshi, Prashant Chaware, Bertha A Rathinam

**Affiliations:** 1 Radiology, All India Institute of Medical Sciences, Bhopal, IND; 2 Anatomy, All India Institute of Medical Sciences, Bhopal, IND; 3 Orthopaedics, All India Institute of Medical Sciences, Bhopal, IND

**Keywords:** : anatomic, asian, femoral condyle, knee, morphometric analysis, tibial condyle

## Abstract

Background

The asymmetric medial and lateral condyles of the distal femur and proximal tibia have a direct influence on the biomechanics of knee joint and prostheses design. This study aimed to determine the morphologic data, that is., anteroposterior (AP) and mediolateral (ML) widths, and the radius of curvature (ROC) of the geometric arcs of the distal femur and proximal tibia.

Methods

One hundred and seventeen adult dry bones (57 femurs and 60 tibias) were studied. Aspect ratios (AP/ML) were calculated. The AP and ML widths were measured using digital Vernier Caliper with a measuring range of 0-150 mm, resolution of 0.01 mm, and accuracy ± 0.02 mm. The geometric arcs of femoral and tibial condyles were divided into three parts namely anterior 1/3rd, distal (femur) or middle (tibia) 1/3rd and posterior 1/3rd and were estimated in the sagittal plane for the femur and transverse plane for tibia using the ROC gauges.

Results

For the femur, the mean AP length for medial and lateral condyles was 55.62 mm and 57.93 mm, respectively, while the mean ML width was 73.45 mm. For the tibia, the mean AP length for medial condyle (MC) and lateral condyle (LC) was 47.74 mm and 43.46 mm, respectively. The mean aspect ratios for the distal femur and proximal tibia were 1.26 and 1.45, respectively. The mean aspect ratios for MC and LC of the femur were 0.50 and 0.52, respectively, whereas, for tibia, they were 0.61 and 0.71, respectively. The mean ROC for femoral MC - 20.77 mm, 31.42 mm, and 19.68 mm and for LC - 21.48 mm, 64.40 mm and 19.06 mm for the anterior, distal and posterior arcs, respectively. The mean ROC for tibial MC - 22.42 mm, 22.49 mm and 19.94 mm, and LC - 19.92 mm, 21.79 mm and 20.95 mm for the anterior, middle and posterior arcs, respectively.

Conclusions

The morphologic data accumulated in this study for both the distal femur as well as the proximal tibia would provide guidelines and help the manufacturers of joint prostheses to address the potential for compromised implant fit and re-design and make available ‘anatomic’ knee prostheses appropriate for the local population which would not only improve function but also prolong the longevity of the prostheses.

## Introduction

Knowledge of the three-dimensional geometry of the knee joint - the distal end of the femur and the proximal end of the tibia - is important as this joint is frequently affected in trauma, primary tumours of bone, metabolic bone disorders and degenerative joint diseases.

The distal end of the femur is widely expanded as a bearing surface for transmission of weight to the tibia. The medial and lateral condyles are significantly asymmetric in their morphology, the lateral condyle being longer anteroposteriorly, flatter and lying at a higher plane. These differences are important determinants of knee joint motion. The proximal surface of the tibia, also called the tibial plateau, is reciprocally expanded and presents medial and lateral articular surfaces for corresponding femoral condyles. The plateau also slopes posteriorly and downwards relative to the long axis of the tibia. The articular surface of the medial condyle (MC) is oval with longer anteroposterior (AP) length while that of lateral condyle (LC) is circular. The menisci which overlie the proximal tibia serve to widen and deepen the tibial articular surfaces that receive the femoral condyles thereby improving the tibiofemoral congruence [1].

Arthroplasty is an effective and routinely performed procedure to relieve pain and improve function in patients with osteoarthritis. Unicondylar knee arthroplasty (UKA) for the treatment of localized symptomatic osteoarthritis [2] while total knee arthroplasty (TKA) for more extensive joint involvement are routine procedures now. Soft-tissue balancing along with optimum coverage of the resected bone surfaces is of prime importance to achieve implant longevity in these procedures. The key dimensions for selecting a suitable implant are based on the patient’s unique anatomy - AP length and mediolateral (ML) width [3,4]. Implant incompatibility has been suggested as a possible reason for poor outcomes following knee replacement surgery [5]. Studies done in the Asian countries have shown differences in morphometric measurements from the Caucasian knees [6-10]. The existing prostheses for these surgeries are designed based on Caucasian morphometric analyses [3,11] and are not always appropriate for the Asian population who have a smaller build and stature when compared with their Western counterparts [6-11] leading to problems of implant size mismatch.

The knowledge of normal morphometry of the femoral and tibial condyles is therefore indispensable in effectively designing these replacement prostheses to be used on the local populations. There are not many published data available on the morphometry of distal femur and proximal tibia from India. Hence, the authors felt the need for a study on the morphometry of the distal femur and proximal tibia.

## Materials and methods

One hundred and seventeen dry adult bones available at two medical institutions in central India were included for the study. Institutional human ethics committee clearances (ref: IHEC-LOP/2014/STS0027) were duly obtained before commencing the study. The bones which exhibited gross pathological changes and manual damages due to storage were excluded. The right and the left-sided bones were identified and sorted. Morphometric features of the distal femur and proximal tibia were measured. The measurement definitions are described in Table 1.

**Table 1:** Measurement definitions for distal femur and proximal tibia. ML: mediolateral; AP: anteroposterior

Measurement definitions for the distal femur	
Bicondylar width (ML width)	Distance between medial and lateral epicondyle
Medial condyle AP length	Distance between most anterior and posterior aspects of the medial condyle
Lateral condyle AP length	Distance between most anterior and posterior aspects of the lateral condyle
Measurement definitions for the proximal tibia	
Bicondylar width (ML width)	Maximum distance between medial and lateral condyles
Medial condyle AP length	Maximum distance between most anterior and posterior aspects of the medial condyle
Lateral condyle AP length	Maximum distance between most anterior and posterior aspects of the lateral condyle

**Figure 1:**
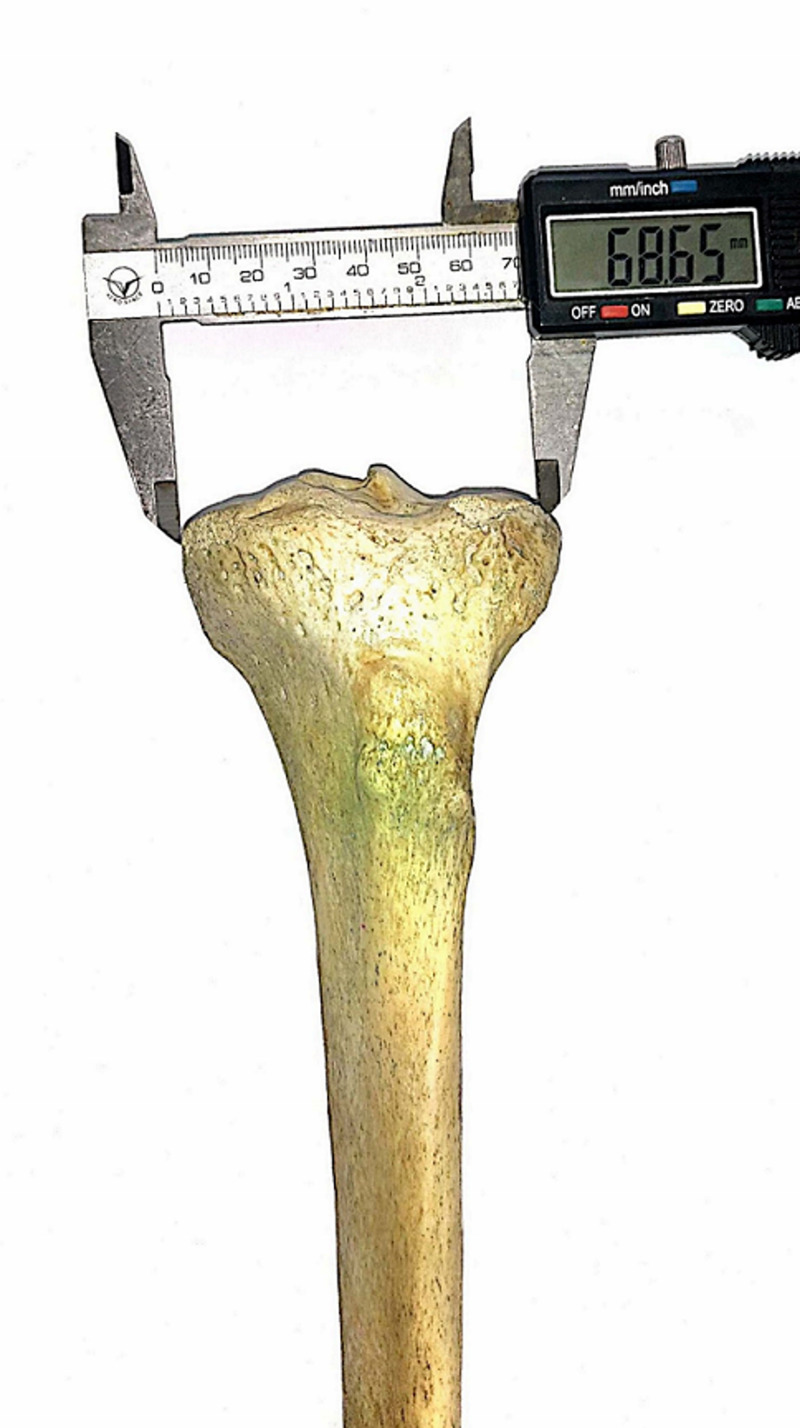
Measurement of bicondylar width of proximal tibia.

All measurements were performed using a digital Vernier Caliper with a measuring range of 0-150 mm, resolution of 0.01 mm, and accuracy ± 0.02 mm (Figure 1). The measurements were made three times by the two of the authors and the mean of the measurements was recorded.

**Figure 2:**
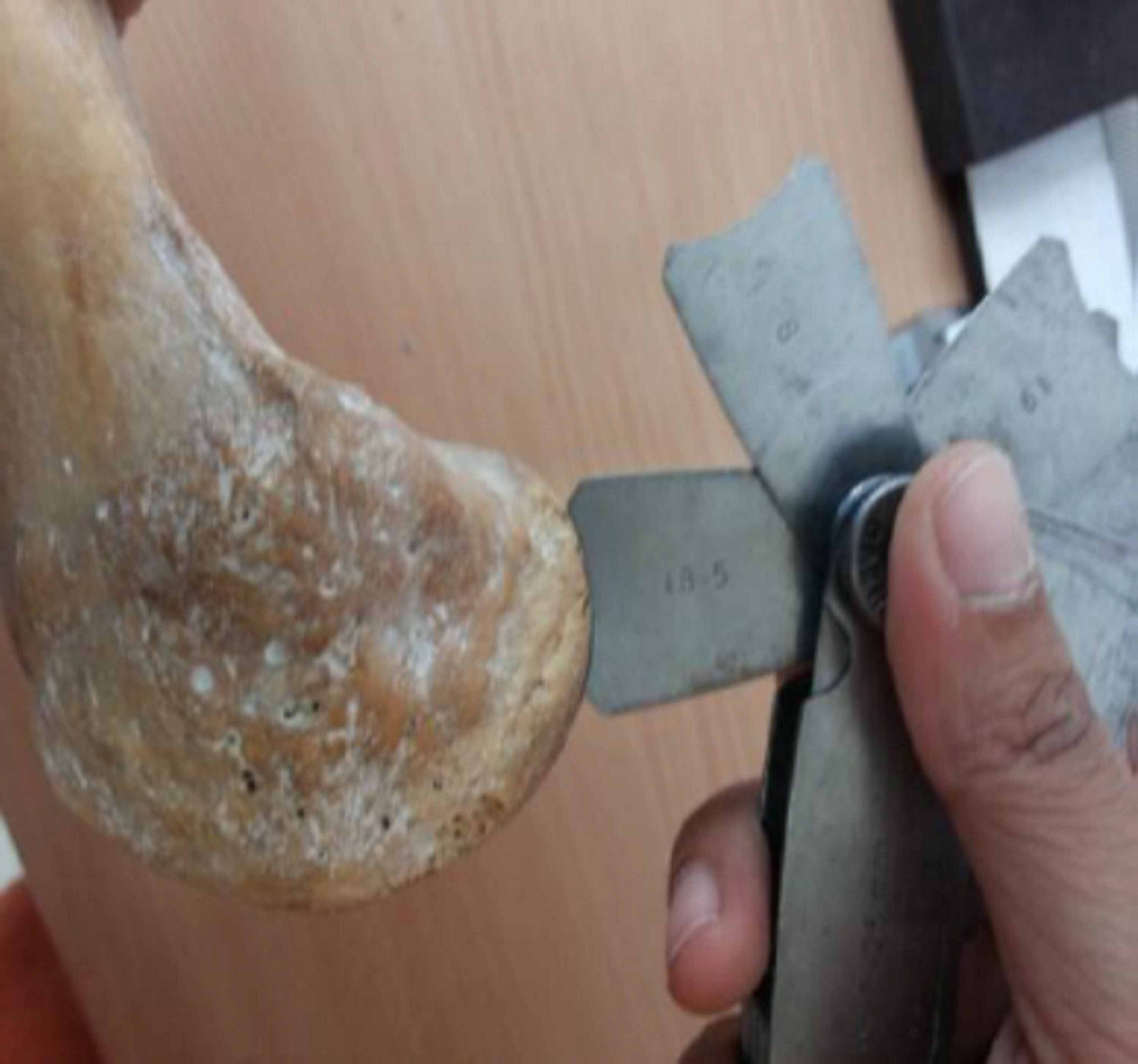
Measurement of the posterior arc of the lateral femoral condyle using the radius of curvature gauge.

**Figure 3:**
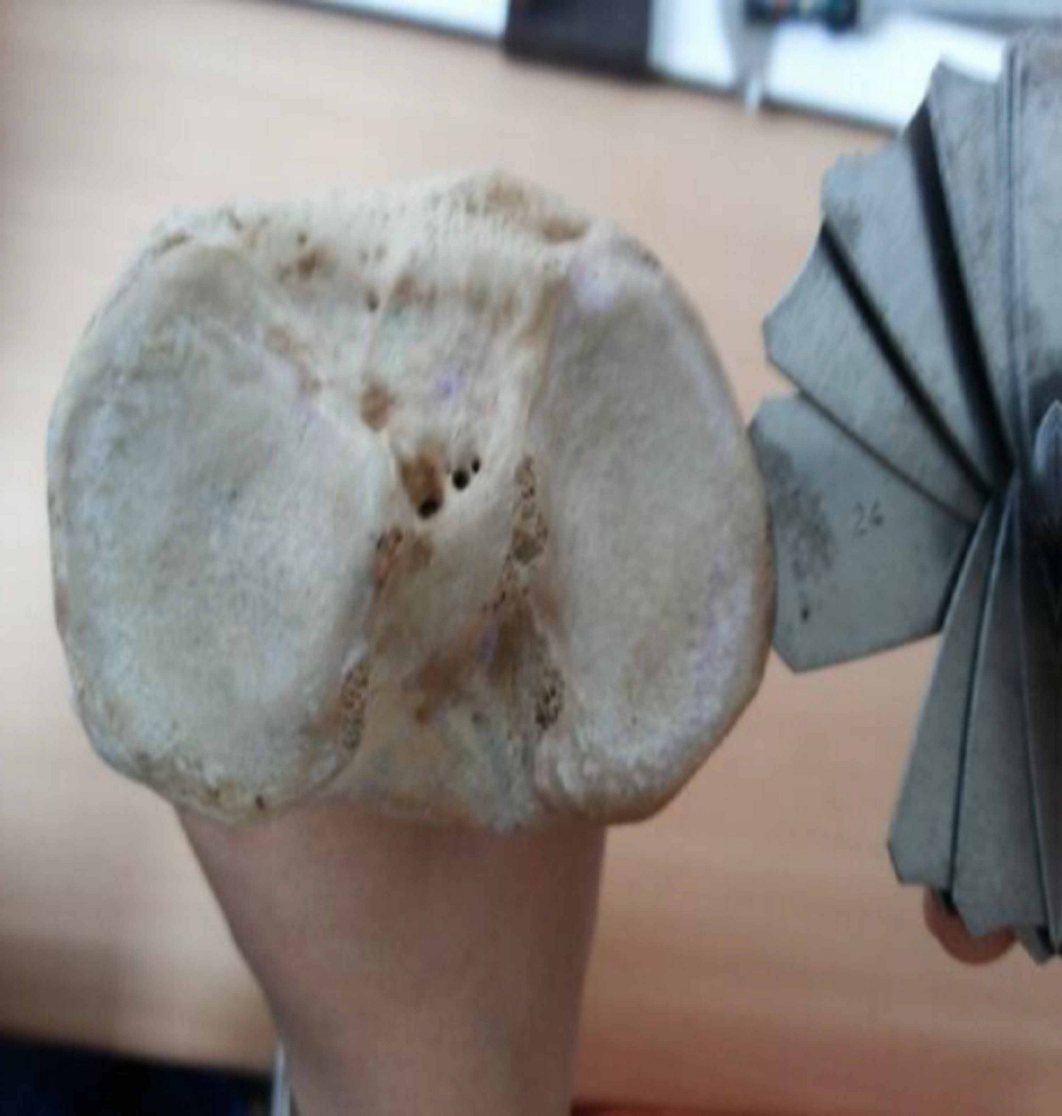
Measurement of middle third arc of tibia.

The geometric arcs of femoral and tibial condyles were divided into three parts namely anterior 1/3rd, distal (femur) or middle (tibia) 1/3rd and posterior 1/3rd and were estimated in the sagittal plane for the femur and transverse plane for tibia using the radius of curvature (ROC) gauges of ranges 7.5-15 mm, 15.5-25 mm, 25.5-35 mm and 35-70 mm (Figure 2, Figure 3).

The measurements were tabulated, and the data were analyzed using the Excel 2010 program (Microsoft®, Redmond, Washington, USA). Mean values of AP length and ML width of femoral and tibial condyles; anterior, distal/ middle and posterior geometric arcs of the same were calculated. Aspect ratios (ratio of ML width to the AP length) for the distal femur and proximal tibia were calculated for each bone and for MC and LC individually.

## Results

Twenty-four femurs (n = 57) and 29 tibias (n = 60) belonged to the right side. Table 2 and Table 3 show the various morphometric measurements of the distal femur and proximal tibia. The mean aspect ratios for the distal femur and proximal tibia were 1.26 and 1.45, respectively. The average aspect ratios for MC and LC of the femur were 0.50 and 0.52, respectively, whereas, for tibia, they were 0.61 and 0.71, respectively.

**Table 2:** Morphometric analysis of the distal femur (n = 57).

Femur	Mean	SD
Length (cm)	43.1	5.1
Antero-Posterior diameter		
Medial condyle (mm)	55.62	4.20
Lateral condyle (mm)	57.93	4.20
Medio-Lateral diameter		
Medial condyle (mm)	28.02	2.77
Lateral condyle (mm)	30.32	2.75
Intercondylar width (mm)	73.45	11.18
Geometric arcs		
Medial condyle		
Anterior – third (mm)	20.77	1.99
Distal – third (mm)	31.42	6.02
Posterior – third (mm)	19.68	1.40
Lateral condyle		
Anterior – third (mm)	21.48	1.49
Distal – third (mm)	64.40	5.88
Posterior – third (mm)	19.06	1.56

**Table 3:** Morphometric analysis of proximal tibia (n = 60).

Tibia	Mean	SD
Length (cm)	37.0	2.5
Antero-Posterior diameter		
Medial condyle (mm)	47.74	3.34
Lateral condyle(mm)	43.46	2.64
Medio-Lateral diameter		
Medial condyle(mm)	30.09	1.81
Lateral condyle(mm)	31.10	2.11
Intercondylar width(mm)	70.66	4.77
Geometric arcs		
Medial condyle		
Anterior – third (mm)	22.42	1.84
Middle – third (mm)	22.49	2.39
Posterior – third (mm)	19.94	4.19
Lateral condyle		
Anterior – third (mm)	19.92	2.65
Middle – third (mm)	21.79	2.17
Posterior – third (mm)	20.95	2.15

## Discussion

Knee replacement is considered a precision surgery, which requires accurate soft tissue balancing and resection of bone thickness equal to the thickness of the prosthetic component implanted, thereby ensuring that the flexion-extension spacing is equal while allowing joint stability throughout the range of motion resulting in a successful outcome [5]. Problems of implant size mismatch are frequently encountered with the existing prostheses as they are designed based on Caucasian morphometric analyses [12] and are not always appropriate for the Asian population who have a smaller build and stature when compared with their Western counterparts [6-10].

The morphometric data obtained in the present study were compared to other studies from Asia retrieved from the PubMed database [5-10,12-17]. Vaidya and colleagues [5] studied computed tomography (CT) of 86 osteoarthritic and 25 dry femurs. In their study, based on CT measurements, the mean AP length was 61.09 mm and 55.58 mm for men and women respectively, while, it was 55.26 mm for the dry femurs. The mean AP length for MC and LC separately were 55.62 mm and 57.93 mm, respectively, in the present study. The mean ML width in their study was 64.84 whereas it was 73.45 mm in the present study. They did not study the proximal tibia. Awasthi and colleagues [12] studied the distal femur in 62 patients presenting with osteoarthritis and trauma of the knee using helical CT, and they reported, the ML width to be 72.74 ± 4.45 mm in males and 63.59 ± 2.61 mm in females, and the AP length to be 49.62 ± 3.86 mm in males and 45.11 ± 4.4 mm in females. Aspect ratio measurements for the distal femur in various studies from Asian countries have ranged from 1.14 to 1.49 [3,6,9,10,15,18]. The mean aspect ratio for distal femur in the present study was 1.26 which is comparable to other studies.

The geometry of proximal tibia has a direct influence on the biomechanics of knee joint [7] and the tibial component is recognized to be more prone to complications compared to the femoral component [2,4]. The aspect ratio of the proximal tibia is an important parameter that helps to anticipate the shape of the tibial component. The aspect ratio in most studies from Asian countries has ranged from 1.33 to 1.8 [7,16,19]. The mean aspect ratio for proximal tibia in the present study was 1.45 which is comparable to other authors as are the aspect ratios for the MC and LC, which were 0.61 and 0.71, respectively [14,20].

This asymmetry of the proximal tibia needs to be incorporated in the TKA prosthesis design as an asymmetric tibial component would be more anatomical, provide correct rotational alignment leading to better survivorship, patellar tracking and function [17,21]. The conventional UKA implants are designed with an asymmetric femoral component and none have an asymmetric tibial component. The present study agrees with Servien and colleagues [20] that the shape of the medial tibial condyle differs from that of the lateral condyle. This difference can lead to ML overhang for medial UKA if the surgeon aims for optimal AP coverage. Custom/patient-specific implants are suggested as they would provide the potential for complete cortical rim coverage better anatomical fit to maximize bone coverage and bone preservation [22,23].

Most authors have only looked at the AP and ML dimensions for the distal femur and proximal tibia [5,6,12,24]. The ROC of the femoral condyles and proximal tibia are not uniform and are integral in dictating normal knee motion as the curvature of the condyles is one of the main factors affecting knee kinematics. In general, a more curved knee would have a higher range of motion [19]. Measurement of the ROC of the femoral condyles is important as the Asian TKA as well as UKA prostheses need to be designed keeping the Asian patient in mind. The ROC of the condyles also has implications for osteochondral allografting procedures [25]. The social and religious needs of prospective patients to keep the knees flexed or for low-sitting, which is a common practice in most Asian cultures, must be incorporated in the prosthesis design [26]. This information is also required for planning the patellar component to avoid problems associated with patellar maltracking.

Various authors have studied the ROC of distal femoral condyles using a variety of techniques. Kapandji [27] divided the medial and lateral femoral condyles into multiple arcs; he noted that posteroanterior the ROC for the medial femoral condyle ranged from 17 to 38 mm and lateral femoral condyle ranged from 12 to 60 mm. Other authors have divided the geometric arcs of the femoral condyles into posterior, distal and anterior arcs. Nuno and Ahmed [28] from Canada studied the profile measurements of the articular surfaces of the femoral condyles in 12 fresh-frozen knees using the two-circular-arc model. They found the posterior arc of the medial and lateral condyle to be 18.9 mm and 19.6 mm respectively and the distal arc measurements as 35 mm and 36.6 mm respectively; in the present study, the mean measurement of the medial and lateral condyle, the posterior arc was 19.68 mm and 19.06 mm respectively and the distal arc was 31.42 mm and 64.4 mm respectively. A smaller ROC was found for the medial condyle than for the lateral condyle; the lateral condyle was much flatter compared to the medial condyle. These observations are comparable to other studies which have noted that the ROC of the posterior arc of lateral condyle is smaller than the anterior and distal arcs [11,19,29].

The measurements of the ROC of the arcs of the proximal tibia would be of immense help in designing tibial components for TKA as well as for UKA; we could not find other researchers looking at this important anthropometric parameter. In the present study, the mean ROC for tibial MC was 22.42 mm, 22.49 mm and 19.94 mm, and for LC 19.92 mm, 21.79 mm and 20.95 mm for the anterior, middle and posterior arcs, respectively.

Some previous studies have used specimen resected during surgery for anthropometric measurements [18,24]. The specimens obtained during knee arthroplasty surgery are obviously arthritic and may not be suitable for basing implant design. We have used dry bones most of which were not arthritic. This makes our data suitable for designing replacement prostheses.

The study has a few limitations. Since we were studying dry bones we were not able to calculate the dimensions for male and female gender separately. Bones in females are generally smaller in dimensions compared to males, and the smaller sized prostheses are generally used in females. Also, the bones were devoid of hyaline cartilage. It has been suggested in previous studies, that the articular cartilage follows the surface topography of subchondral bone, and measurements of the sagittal radii can be extrapolated from the bone measurements [30]. Hence, it may be assumed that the shape and dimension of the distal femur and proximal tibia measured along the periphery are impacted little by the thickness of the articular cartilage [19]. The bone samples, in the present study, were only from central India. To make this study pan-Asian, we would suggest researchers in other countries to undertake similar anthropometric measurements so that we get reliable data available from all over Asia. Such collated data would help in designing UKA and TKA prostheses for our patients.

## Conclusions

The morphologic data accumulated in this study for both the distal femur as well as proximal tibia would provide guidelines and help the manufacturers of joint prostheses to address the potential for compromised implant fit and re-design and make available ‘anatomic’ knee prostheses appropriate for the Asian knee which would not only improve function but also prolong the longevity of the prostheses. 
